# The fungicide triadimefon affects beer flavor and composition by influencing *Saccharomyces cerevisiae* metabolism

**DOI:** 10.1038/srep33552

**Published:** 2016-09-15

**Authors:** Zhiqiang Kong, Minmin Li, Jingjing An, Jieying Chen, Yuming Bao, Frédéric Francis, Xiaofeng Dai

**Affiliations:** 1 Institute of Food Science and Technology, Chinese Academy of Agricultural Sciences/Key Laboratory of Agro-Products Processing/Laboratory of Agro-products Quality Safety Risk Assessment, Ministry of Agriculture, Beijing 100193, P. R. China; 2 Functional and Evolutionary Entomology, Gembloux Agro-Bio-Tech, University of Liège, Passage des Déportés 2, 5030 Gembloux, Belgium; 3 College of Food Science, Northeast Agricultural University, Key Laboratory of Dairy Science, Ministry of Education, Harbin 150030, China

## Abstract

Despite the fact that beer is produced on a large scale, the effects of pesticide residues on beer have been rarely investigated. In this study, we used micro-brewing settings to determine the effect of triadimefon on the growth of *Saccharomyces cerevisiae* and beer flavor. The yeast growth in medium was significantly inhibited (45%) at concentrations higher than 5 mg L^−1^, reaching 80% and 100% inhibition at 10 mg L^−1^ and 50 mg L^−1^, respectively. There were significant differences in sensory quality between beer samples fermented with and without triadimefon based on data obtained with an electronic tongue and nose. Such an effect was most likely underlain by changes in yeast fermentation activity, including decreased utilization of maltotriose and most amino acids, reduced production of isobutyl and isoamyl alcohols, and increased ethyl acetate content in the fungicide treated samples. Furthermore, yeast metabolic profiling by phenotype microarray and UPLC/TOF-MS showed that triadimefon caused significant changes in the metabolism of glutathione, phenylalanine and sphingolipids, and in sterol biosynthesis. Thus, triadimefon negatively affects beer sensory qualities by influencing the metabolic activity of *S. cerevisiae* during fermentation, emphasizing the necessity of stricter control over fungicide residues in brewing by the food industry.

*Saccharomyces cerevisiae* is a yeast species widely used for fermentation in winemaking, baking, and brewing since ancient times. It is well known that *S. cerevisiae* produces different concentrations of aroma compounds as a function of fermentation conditions and medium treatments^1^. Additionally, simply changing the fermentation medium composition significantly affects aroma compound synthesis^2^. Barley, the major raw material used in brewing, determines the beer quality. However, because of their high starch and storage protein contents, barley grains represent an attractive source of nutrients for microbial pathogens^3^. Therefore, fungicides are widely used in different combinations at many stages of barley cultivation and during post-harvest storage^4^.

Triadimefon [1-(4-chlorophenoxy)-3,3-dimethyl-1-(1H-1,2,4-triazol-1-yl)-2-butan-one] (TF) is a systemic wide-spectrum antifungal compound that belongs to the group of sterol biosynthesis-inhibiting fungicides, which interfere with the formation of fungal cell walls^5^. TF is the main pesticide used during cultivation to protect barley from diseases infestation^6^,^7^. Although precautions are taken to limit the harmful effects of pesticide use, residues/metabolites on barley grain may be transferred to the wort and persist during brewing, with the possibility of exerting potentially deleterious effects on beer quality. Miyake *et al*.^8^ have found that a high proportion of fungicide residues are carried over to young beer. Some pesticides have been shown to inhibit the growth and metabolic activity of yeasts, leading to delayed or arrested fermentation^9^, and this will affect color and flavor of the end product^10^. Therefore, it is important to determine the effect of TF on yeast activity during brewing to ensure the quality of beer.

The sensory quality of beer is important to gain the consumer’s attention. Higher alcohols and esters produced during fermentation significantly contribute to the flavor character of beer by supplying specific fruity and floral aroma, and their content is carefully monitored by beer producers^11^,^12^. As stated above, pesticide residues may have remarkable antiseptic activity on the yeasts and other microorganisms (such as bacteria), thereby leading to delayed or stuck (incomplete and arrested) fermentation^9^. Navarro *et al*.^13^ have found that fungicides affect the fermentation rate, decreasing color intensity and increasing the tint of young lager beer. However, most studies investigated the effects of pesticides on the quality of wines^14^,^15^,^16^,^17^, while significantly less attention has been paid to beer^9^. As a result, little information is currently available regarding pesticide effects on the sensory quality of beer.

Phenotype microarray (PM) technology and advances in mass spectrometry (MS) technology allow large-scale metabolic profiling of microorganism systems. Metabolomics is an emerging “omics” domain that investigates the presence of small organic molecules (endogenous metabolites) in the cell, known as the metabolome^18^. Metabolomics allows studying differences in the metabolome as a result of exposure to a toxicant and linking of the specific metabolites formed to biochemical and functional processes in order to gain insight in the pathways underlying the toxic effects^19^. The PM technology enables monitoring cellular responses to various environmental challenges using a specific reporter system^20^. Liquid chromatography–mass spectrometry (LC–MS)-based approaches are expected to be of particular importance in metabolomics analyses, largely because of the widespread availability of the technology and the ready compatibility of broad separations with biological samples^21^. Furthermore, ultra-performance liquid chromatography (UPLC)–MS is considered the ideal technique for metabolite profiling owing to its high reproducibility of retention time and reduced ion suppression^22^,^23^. The use of metabolomics to understand the interplay effects of pesticides on fermenting yeasts is of paramount importance^9^. Thus, the combined use of PM- and UPLC–MS-based metabolomics approaches enables elucidating the pathways that are influenced by pesticides.

The objective of the present work was to evaluate whether the presence of TF can negatively affect yeast growth and the sensory quality of beer by influencing *S. cerevisiae*. The sugars and the amino acids as precursors of flavor substances were determined and different types of higher alcohols and esters were investigated in a micro-brewing system. Furthermore, we investigated the biochemical changes in *S. cerevisiae* treated with and without TF by combining PM- and UPLC-MS-based metabolomics approaches. Potential biomarkers related to the metabolic pathways were identified to better understand the effect of TF on *S. cerevisiae* during brewing.

## Results and Discussion

### Effect of TF on *S. cerevisiae* growth

**Figure 1:**
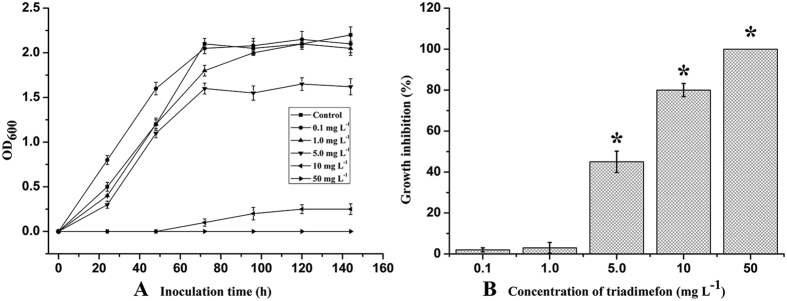
Effect of TF on *S. cerevisiae* growth in malt extract. (**A**) Growth curves of *S. cerevisiae* in malt extract containing different concentrations of TF. (**B**) Inhibition of growth parameters by triadimefon. The data are expressed as the mean ± SD (n = 3), an asterisk indicates a significant difference versus the control (*P* < 0.05).

The analysis of *S. cerevisiae* growth curves indicated that at low concentrations (0.1 and 1.0 mg L^−1^), TF did not significantly affect yeast growth as compared to the untreated control, and even stimulated growth (0.1 mg L^−1^) from 0 to 72 h (Fig. 1A). This was probably because TF provided an additional source of nitrogen in the medium, facilitating yeast proliferation; these results are consistent with those of Navarro *et al*.^24^. As shown in Fig. 1B, yeast growth was not significantly inhibited by TF at concentrations ≤1.0 mg L^−1^; only 1.8% and 3.1% growth inhibition was observed in the presence of 0.1 mg L^−1^ and 1.0 mg L^−1^, respectively. At concentrations >5.0 mg L^−1^, TF significantly reduced the colony-forming ability, reflective of cell stress (45%, 80%, and 100% growth inhibition at 5, 10, and 50 mg L^−1^, respectively). Based on these results, 5 mg L^−1^ TF was used for malt fortification to evaluate its influence on beer fermentation.

Our results indicated that the efficiency of malt fermentation can be expected to significantly decrease in the presence of high TF contamination. Pesticide residues, especially fungicide residues, are one of the main factors influencing the occurrence and growth of yeasts during fermentation^25^,^26^. Multiple studies have reported the effects of herbicides on yeast biological parameters. Herbicide treatments are able to affect yeast growth by affecting the enzymatic activity of catalase and superoxide dismutase, as well as inducing oxidative modifications of proteins^27^.

### Responses of the ET and EN to beer fermented in the presence or absence of TF

The human tongue and nose remain the most effective tools for the identification and characterization of food flavor, and sensory panels are an integral part of the quality control process in the food industry; however, they are expensive, and training and analysis are also time-consuming. The multisensory ET and EN systems present effective alternative approaches to taste evaluation and have been successfully applied in the food industry^28^,^29^,^30^,^31^.

Figure S1A shows typical responses of the ET sensors, where each curve presents the kinetics of the sensor potential (μs cm^−1^). Significant changes were observed during the first 10 s after which a dynamic balance was achieved for all sensors, except UMS. Therefore, ET signals at 120 s were used for sample comparison. Figure S1B shows that the responses of the EN sensors reached a dynamic balance after 20 s, and the signals at 60 s were selected for comparison. The stability of sensor responses (ET at 120 s and EN at 60 s) was analyzed by calculating the relative standard deviation (RSD). Only sensors with good stability (small RSD, <15.0) in the target matrix are to be included in the sensor array used for evaluation^30^,^32^. In our experiments, each sensor exhibited high stability (Table S1). The results of one-way ANOVA for 17 sensors demonstrated significant differences between beers treated or not with TF (*P* < 0.05; Table S1).

**Figure 2:**
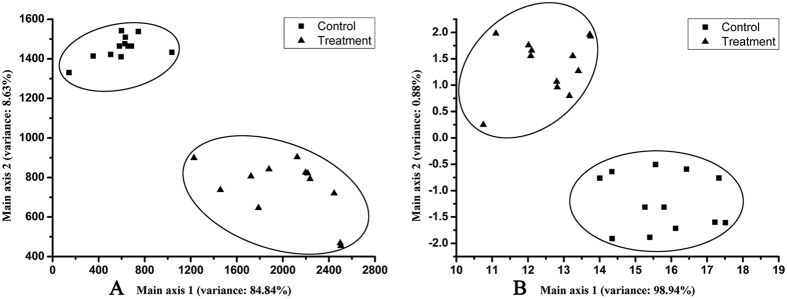
PCA of beer samples tested by the electronic tongue (A) and electronic nose (B). The percentage of total variance covered by principal components is indicated on the axis.

The results of PCA indicated that the ET and EN could effectively discriminate beers with and without TF (Fig. 2). The first two PCs (PC1 and PC2) could explain 93.47% and 99.82% of the total system variance (Fig. 2A,B, respectively). All beer samples were clearly differentiated in the PCA plot, which demonstrated that the same types of beer samples were clustered together. The PCA revealed that TF affected beer flavor and quality, also indicating potential food safety problems, as evidenced by the TF effect on yeast growth.

### TF residue effects on beer carbohydrate content

The most important process in the transformation of wort into beer is the alcoholic fermentation, in which sugar is transformed into ethanol and carbon dioxide by yeast enzymes. Sugar contents (g L^−1^) in the malt extract were as follows: maltose, 29.6; maltotriose, 8.7; glucose, 7.8; fructose, 1.2; and sucrose, 1.1. Under normal conditions, the yeast utilizes wort sugars in the following order: sucrose, glucose, fructose, maltose, and maltotriose. To achieve an efficient conversion of sugars to ethanol (good attenuation), the yeast should be able to use maltose and maltotriose, the major sugars in brewing malt.

At the end of the fermentation, only residual glucose, maltose, and maltotriose were found in both the control and the TF-treated beer samples (Table 1). Fructose and sucrose were completely metabolized after fermentation in all samples. Sucrose has to be split into fructose and glucose by the yeast-produced enzyme invertase, prior to being assimilated by the yeast^33^. There were no significant differences between the treatment groups in glucose and maltose content at the end of fermentation. However, the concentration of maltotriose was significantly higher after TF treatment (1.8 g L^−1^) than in the controls (0.6 g L^−1^), which is consistent with previous findings that other fungicides inhibiting sterol biosynthesis, such as propiconazole, diniconazole, and epoxiconazole, influenced yeast consumption of fermentable carbohydrates, especially, maltose and maltotriose^13^,^24^.

### Effects of TF on the content of higher alcohols and esters

Higher alcohols and esters found in alcoholic beverages are secondary metabolites produced by *S. cerevisiae* during fermentation^34^ and are the major contributors to the flavor of fermented beverages^35^,^36^. A total of five higher alcohols and seven higher esters were identified in beer samples with or without TF, and the concentrations of isobutyl and isoamyl alcohols and ethyl acetate were significantly different between both treatment groups (*P* < 0.05; Table 2). Lower concentrations of isoamyl and isobutyl alcohols in the fungicide-treated samples may be due to decreased assimilation and/or biosynthesis of the alcohol precursors leucine and valine, respectively, by *S. cerevisiae*^37^. Similar results were obtained for grapes treated with insecticide chlorpyrifos^38^. TF seemed to promote ethyl acetate synthesis, as evidenced by its significantly higher concentration in TF-treated than in untreated beers (34.5 mg L^−1^ vs. 22.3 mg L^−1^). Kagan *et al*.^39^, who studied changes in gene expression of *S. cerevisiae* after treatment with ergosterol biosynthesis inhibitors, found that *ATF2*, encoding an alcohol acetyl transferase, was upregulated by TF. Further, the expression levels of *ATF2* greatly enhanced the production of ethyl acetate during fermentation^40^.

### Effects of TF on amino acid content

Amino acids essential to yeast growth and metabolism during alcoholic fermentation^41^ are known to affect the synthesis of higher alcohols and esters in fermented beverages^42^. Therefore, to confirm the effect of fungicide residues on the flavor and quality of beer, we examined amino acid content in the experimental samples by automatic amino acid analyzer.

**Figure 3:**
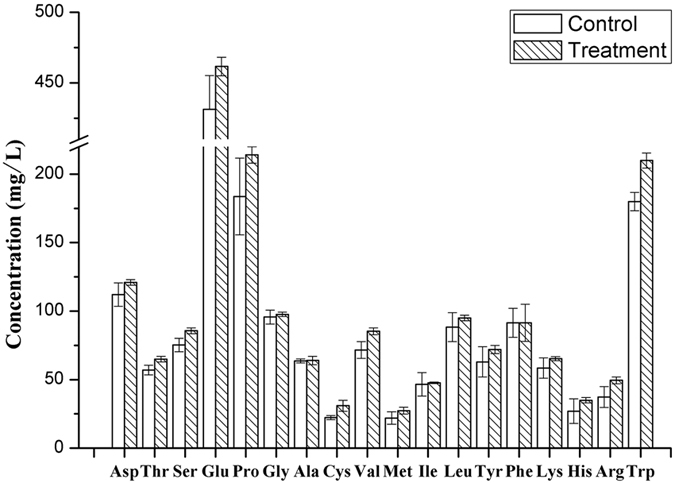
Amino acid yields at the end of fermentation.

The chromatograms of amino acid standards and beer samples are shown in Fig. S2, and the amino acid profiles of beer fermented with or without TF are shown in Fig. 3. The concentrations of most amino acids (except for alanine, isoleucine, and phenylalanine) were higher in the fungicide-treated than in the control beer. Amino acids in the untreated beer were most likely transformed into their corresponding metabolites during fermentation, as was observed during the production of apple cider^43^. However, given that yeasts utilize amino acids as a nitrogen source for the synthesis of proteins and other cellular components^44^, TF seems to suppress yeast metabolic activity and arrest fermentation. The finding that TF-treated beers had lower concentrations of isoamyl and isobutyl alcohols produced from precursor amino acids leucine and valine, respectively, further supports the conclusion that TF residue decreased beer quality. The observation that beers with high concentrations of residual amino acids have a greater risk of microbiological instability with the possible formation of biogenic amines and ethyl carbamate, which have a negative impact on beer quality, corroborates this^45^.

### Analysis of *S. cerevisiae* metabolism using the PM assay

We evaluated the effect of TF treatment on *S. cerevisiae* metabolic potential, which enabled us to speculate on possible effects of TF on beer quality. PM analysis was performed in a set of eight 96-well microplates (PM1 to PM8) containing different nutrients. PM1 and PM2 contained 190 alternative carbon sources; PM3, 6, 7, and 8 contained 380 alternative nitrogen sources; and PM4 and PM5 contained phosphorus, sulfur, and other nutrients; end-point measurement of cell respiration was based on the reduction of a tetrazolium dye. This design allowed testing 768 cellular phenotypes in a sensitive, highly controlled, and reproducible format. The OmniLog system captured digital images of the MicroArray four times per hour and converted the quantitative values into kinetic graphs.

The results presented in Fig. S3 revealed marked differences between TF-treated and untreated yeast. *S. cerevisiae* exposed to the fungicide showed slower growth on carbon sources such as maltotriose (PM1: E10) and nitrogen sources such as l-leucine, l-tyrosine, l-valine, and guanine (PM3B: B5, C1, C2, and F6, respectively), which is consistent with the results on beer fermentation. TF-treated yeast also showed a reduced ability to utilize phosphorus and sulfur sources (PM4A: B12, C1, C7, C8, D12) and other nutrients (PM5: G6, G7, G8; PM7: A5). Deregulation of these metabolic pathways may result in a change of beer flavor, which should be further studied during fermentation. However, TF-treated *S. cerevisiae* was significantly more active in using small peptides as nitrogen sources (PM6: G7) as compared to control yeasts, which may be due to the enhancement of yeast metabolism as an adaptive response to xenobiotic stress.

### Metabolomics and metabolic pathway analysis

**Figure 4:**
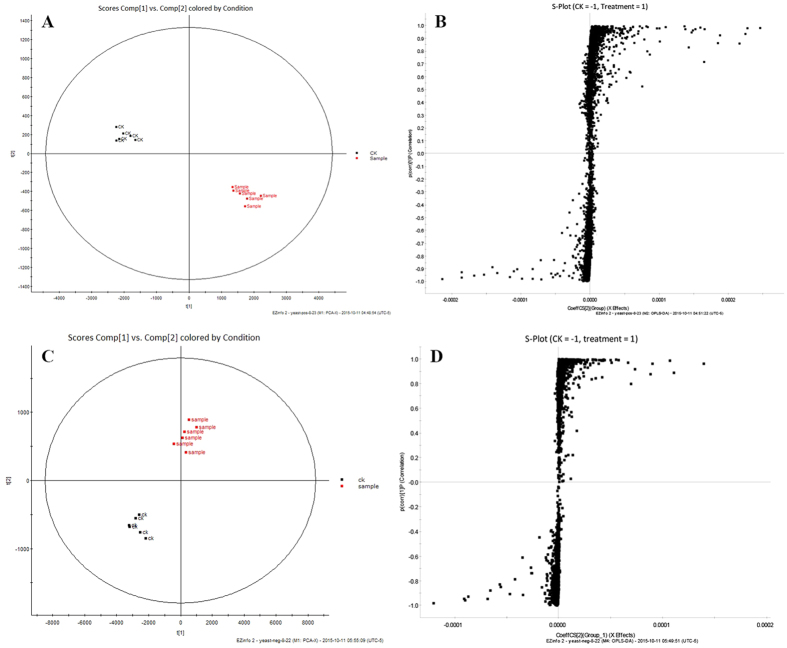
Metabolomic profiling of *S. cerevisiae* incubated with TF by UHPLC/TOF-MS. (**A**) OPLS-DA score map of the extracts of TF-treated and untreated *S. cerevisiae* in the positive ionization mode (ESI^+^), with an accuracy of 98.5%, multiple correlation coefficient (R^2^) of 91.7%, and cross-validated R^2^ (Q^2^) of 87.3%. (**B**) Representative S-plot of TF-treated (+1) versus and untreated (−1) *S. cerevisiae* in ESI^+^. (**C**) OPLS-DA score map of the extracts of TF-treated and untreated *S. cerevisiae* in the negative ionization mode (ESI^−^), with an accuracy of 93.2%, R^2^ of 90.1%, and Q^2^ of 78.4%. (**D**) Representative S-plot of TF-treated (+1) and untreated (−1) *S. cerevisiae* in ESI^−^.

To confirm the results obtained by the PM assay, we compared metabolic profiles of *S. cerevisiae* treated or not with TF, using multiple pattern recognition methods. OPLS-DA and S-plots were applied to classify metabolic phenotypes and identify metabolites affected by TF treatment. In the OPLS-DA score plot, the biological replicates of the treatment groups were effectively clustered, separating the control from fungicide-treated yeasts (Fig. 4A,C). MS signals responsible for this differentiation were characterized by VIP from the corresponding S-plots generated by OPLS-DA (Fig. 4B,D): the farther away from the origin, the higher the value of the ions in the VIP. Potential markers were extracted from the S-plots, and markers were selected according to their contribution to the variation and correlation within the data set. Based on the S-plot and corresponding loading plot, differences between the control and TF-treated yeasts were detected for 20 metabolites in the positive ion mode and for 11 metabolites in the negative ion mode (Table S2). The molecular mass was determined within a reasonable degree of measurement error using UPLC/TOF-MS, and the potential element composition, degree of unsaturation, and fractional isotope abundance of the compounds were screened against the ChemSpider (http://www.chemspider.com/), Human Metabolome Database (http://www.hmdb.ca/), and other databases to determine chemical composition.

**Figure 5:**
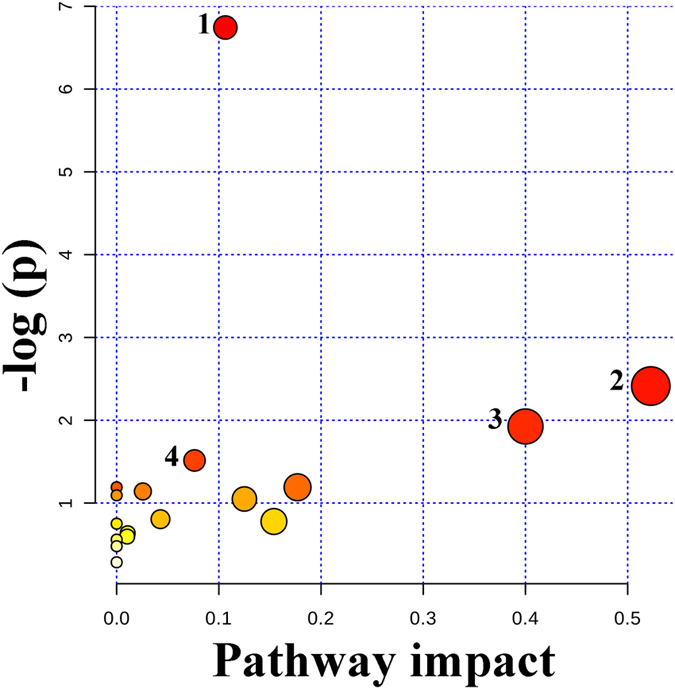
Summary of ingenuity pathway analysis based on KEGG data. The matched pathways are presented as circles. The color intensity and size of each circle are based on *P*-value and pathway impact value, respectively; darker color reflects more significant coordinated metabolic changes in the corresponding pathway, while bigger circle size (corresponding to pathway impact score) reflects higher centrality of the involved metabolites. The x-axis indicates the relative between-ness of the quantified metabolites in their relevant metabolic pathways. (1) Steroid biosynthesis, (2) glutathione metabolism, (3) phenylalanine metabolism, (4) sphingolipid metabolism.

To clarify whether the observed changes in metabolite content correlated with those in corresponding metabolic pathways, we performed pathway analysis with MetaboAnalyst 3.0^46^, which employs data from the KEGG PATHWAY Database. Detailed evaluation of metabolic pathways revealed that four pathways were significantly perturbed in TF-treated yeasts: steroid biosynthesis, glutathione metabolism, phenylalanine metabolism, and sphingolipid metabolism (Fig. 5). TF inhibited the synthesis of ergosterol, the major sterol component in the fungal membrane critically important for its structure and function, thus negatively affecting yeast fermentation activity. The glutathione metabolic pathway restores intracellular GSH/GSSG balance and contributes to cell protection against oxidative stress^47^. TF down regulated phytosphingosine involved in sphingolipid metabolism, which plays a vital role in the regulation of cellular transport, protein function, and signal transduction^48^. Although TF did not change the level of phenylalanine in *S. cerevisiae*, it negatively affected phenylalanine metabolism, as well as lysine degradation and biosynthesis, and metabolism of histidine, tryptophan, cysteine, and methionine. Together, these data indicated that TF acts mainly by influencing steroid biosynthesis to inhibit cell wall synthesis, so as to reduce the fermentation activity of yeast, furtheraffecting other metabolite pathways to impact the synthesis of flavor compounds.

## Conclusions

In this work, we examined the influence of the fungicide TF on the quality of beer by determining the sugar, amino acid, and volatile compound contents and evaluating sensory characteristics by using electronic tongue and nose. While at low concentrations, TF did not significantly affect the yeast growth, its presence in malt extract at 5 mg L^−1^ inhibited yeast growth by 45%. TF treatment of *S. cerevisiae* compromised the fermentation of maltotriose and utilization of most amino acids, which resulted in the reduction of isobutyl and isoamyl alcohol and induction of ethyl acetate synthesis during fermentation. Metabolic profiling by PM and UPLC/TOF-MS revealed that TF treatment significantly influenced steroid biosynthesis and the metabolism of glutathione, phenylalanine, and sphingolipid, closely related to yeast fermentation activity. Our findings suggest that the presence of residual TF might negatively affect *S. cerevisiae* fermentation activity, which in turn influences the flavor and quality of the resultant beer.

## Materials and Methods

### Chemicals and reagents

TF (98%) was purchased from Dr. Ehrenstorfer (Augsburg, Germany). HPLC-grade acetonitrile, methanol, and trichloromethane were purchased from Fisher Chemicals (Pittsburgh, PA, USA), and formic acid was obtained from Dikma Technologies (Lake Forest, CA, USA). Amino acid standards, ninhydrin, and leucine-enkephalin were supplied by Sigma-Aldrich (St. Louis, MO, USA). Standards of higher alcohols and esters were purchased from AnPu Company (Shanghai, China), and those of glucose, fructose, sucrose, maltose, and maltotriose (all >99% purity) were obtained from Dr. Ehrenstorfer. Hydrochloric acid, phenol, and sodium citrate for the analysis of amino acids were of analytical grade (Beijing Chemical Reagent, Beijing, China). Phosphate-buffered saline (PBS) was from Gibco BRL (Gaithersburg, MD, USA). HPLC-grade water was produced using an Arium comfort I ultra-pure water system (Sartorius A. G., Göttingen, Germany).

### Evaluation of the effect of TF on yeast growth

Dry *S. cerevisiae* CICC 1202 (China Center of Industrial Culture Collection, Beijing, China) was rehydrated, inoculated on Yeast Nitrogen Base (YPD)-agar plates (10 g L^−1^ yeast extract, 20 g L^−1^ peptone, 20 g L^−1^ glucose, and 20 g L^−1^ agar), incubated at 30 °C for 24 h, and kept at 4 °C until use.

For the experiment, a single colony was inoculated into YPD medium and grown overnight at 30 °C with agitation. Then, the culture was diluted with fresh YPD medium to OD_600_ = 0.1 and grown to OD_600_ = 1.0. Yeast cells were harvested by centrifugation (5,000 rpm, 10 min, 4 °C), washed twice with 50 mM PBS, resuspended at 2.5–5.0 × 10^5^ CFU mL^−1^ in 200 mL malt extract broth supplemented or not with TF (0.1, 1, 5, 10, and 50 mg L^−1^), and grown at 30 °C with agitation (120 rpm) for 5 h. Samples were taken at the indicated time intervals to determine the OD_600_ using an Evolution 260 Bio UV-visible spectrophotometer (Thermo Fisher Scientific, Waltham, MA, USA) and to construct a *S. cerevisiae* growth curve during fermentation. CFU numbers were determined by using the limited dilution method using triplicate YPD agar plates incubated at 30 °C for 2–3 days.

### Evaluation of the effect of TF on brewing

TF effects on beer quality were investigated using a micro-brewing system containing 2.0 kg of malt extract mixed with 20 L water and 5.0 μg mL^−1^ TF (control sample was left without TF); the malt extract (the concentration of α-N was 180.5 mg/L at 12 degrees Plato (°P, specific gravity as the weight of extract in 100 g of solution, at 17.5 °C)) and the ingredients that were fed into the brewing system were purchased from Jiefei food Co., Ltd. (Dalian, China), the ingredients were evenly stirred and yeast was sprinkled over the top of the mixture. After 168-h fermentation at 24 °C, the beer was evaluated for flavor, and organic content. Three independent brewing experiments were performed with both control and fungicide treatment.

### Sensory evaluation

Taste sensor: An electronic tongue (ET; Alpha M.O.S., Toulouse, France) was used to measure the electric potential of the young lager beer. The analyzer consisted of seven liquid cross-sensitive sensor electrodes (SRS, GPS, STS, UMS, SPS, SWS, and BRS), which had an organic coating responsible for the sensitivity and selectivity of the sensor. The electric potential difference between each sensor and the Ag/AgCl reference electrode was measured at the equilibrium state at room temperature, and the signal from each sensor was integrated to determine sample taste based on 12 measurements (In each independent brewing system, 4 samples were obtained to test for the electronic tongue and nose).

Flavor sensor: An electronic nose (EN; Airsense, Schwerin, Germany) consisted of a sampling apparatus, detector, and pattern recognition software. The detector contained 10 metal oxide semiconductor-type chemical sensors: W1C (aroma), W5S (broad range), W3C (aroma), W6S (hydrogen), W5C (aromatic-aliphatic), W1S (broad methane), W1W (sulfur-organic), W2S (broad alcohol), W2W (sulfur-chlorine), and W3S (methane-aliphatic). The measurement conditions were as follows: sample volume, 1 mL; vial volume, 10 mL; injection volume, 100 μL.

### Measurement of beer organic contents

Sugars: Beer samples were cooled to room temperature and filtered through a 0.22-μm Millipore membrane before analysis. An HPLC system (e2695 series; Waters Corp., Milford, MA, USA) equipped with a refractive index detector was used to simultaneously separate and analyze sucrose, glucose, fructose, maltose, and maltotriose. A 10-μL aliquot of each sample was injected into an Xbridge BEH Amide column (5 μm, 4.6 × 250 mm) kept at 45 °C; the mobile phase consisted of acetonitrile:water (85:15, v/v) containing 0.2% trimethylamine degassed in an ultrasonic bath and used at a flow rate of 1.0 mL min^−1^. For method validation, please refer to the Supplementary Materials.

Higher alcohols and esters: Beer bottles were cooled at 4 °C to prevent the loss of volatile compounds, and 10 mL aliquots of beer or standard solutions were transferred to headspace vials (40 mL) containing 3.0 g NaCl. The vials were sealed with polyethylene and silicone septum caps, and the samples were conditioned in a thermostatic bath at 70 ± 1.0 °C for 1 h. Then, a manual solid-phase microextraction holder with 75-μm-thick carboxen/polydimethylsiloxane fibers (Supelco, Bellefonte, PA, USA) was pierced with the needle, and the fiber was exposed to the headspace for 30 min at 60 ± 1.0 °C.

The extracted analytes were desorbed in the injection port of the GC-MS system (ISQ Single Quadrupole GC-MS, Thermo Fisher Scientific) equipped with a fused silica capillary column (30 m × 0.25 mm inner diameter; film thickness, 0.25 mm) coated with Zebron ZB-Wax (Phenomenex, Macclesfield, UK); injections were performed in the splitless mode. The conditions were as follows: injector temperature, 200 °C; flow rate of the carrier gas (99.999% helium), 0.8 mL min^−1^; column temperature, 40 °C for 4 min, increased by 5 °C per min to 120 °C for 1 min and by 8 °C per min to 220 °C for 2 min. MS analysis was performed in a full-scan mode in the range of 35–450 amu; the ion source was maintained at 200 °C, and ionization energy was 70 eV. Chemicals were identified by comparison of their retention times and mass spectra with those of standard higher esters and alcohols. For method validation, please refer to the Supplementary Materials.

Amino acids: Beer (10 mL) was hydrolyzed with 6 M HCl (10 mL) in a glass tube and 200 μL phenol was added. The tube was frozen in a dry ice-ethanol bath for 5 min and filled with nitrogen to prevent oxidation, evacuated, sealed, and placed in a temperature-controlled oven at 110 °C for 22 h. The mixture was allowed to cool, and the tube was opened using a glass knife. The hydrolysate was filtered, transferred to a 50-mL volumetric flask, and added up to 50 mL with ultrapure water. One milliliter of the solution was transferred to a round-bottom flask and concentrated to near dryness using a rotary evaporator at 50 °C. The residue was reconstituted in 2 mL of ultrapure water, evaporated to dryness, redissolved in 1 mL of sodium citrate buffer (pH 2.2), and the samples were inject into a HITACHI automatic amino acid analyzer (Model: L-8900, Japan) for analysis.

### PM analysis

*S. cerevisiae* CICC 1202 cultures treated or not with 5 mg L^−1^ TF were subjected to full 8-panel PM analysis using the Phenotype MicroArray^TM^ (Biolog Inc., Hayward, CA, USA). Briefly, Biolog growth medium was prepared using 0.67% (w/v) YNB minimal medium supplemented with 6% (w/v) glucose, 2.6 μL of yeast nutrient supplement mixture (48–24 mM adenine-HCl, 4.8 mM l-histidine-HCl monohydrate, 48 mM l-leucine, 24 mM l-lysine-HCl, 12 mM l-methionine, 12 mM l-tryptophan, and 14.4 mM uracil), and 0.2 μL of dye D (Biolog). Yeast cultures were mixed with the medium in the PM plates, which were read at 15-min intervals for 24 h, and kinetic data were analyzed with the OmniLog-PM software. This software generates time course curves for respiration and calculates differences in the areas for control and treatment cells. The differences are averages of values reported for two separate experiments. In all cases, a minimum of three replicate PM assays per plate was conducted, and the average of the signal values was used.

### Metabolomics

Yeast cultures grown from a single colony as described above were treated or not with 5 mg L^−1^ TF for 2 h, centrifuged, washed with PBS, pelleted, mixed with 1 mL methanol at −40 °C, and lyophilized. Cellular metabolites were extracted from 50 mg of dry sample with 1.1 mL of trichloromethane-methanol-formic acid (50:50:0.1) in a 2.0-mL Eppendorf tube, pulverized with 1-mm glass beads, using a FastPrep-24^TM^ 5G homogenizer (MP Biomedicals, Santa Ana, CA, USA) at 6.5 m s^−1^ for 40 s twice, and centrifuged at 12,298× *g* for 10 min at 4 °C. The supernatants were filtered through 0.22-μm filters and then through 10-kDa ultrafiltration membranes to remove cellular debris and residual proteins, and transferred to autosampler vials for UPLC-Q-TOF-MS analysis.

UPLC-Q-TOF-MS was performed using an ACQUITY UPLC-I-Class system coupled with a Xevo G2-XS TOF mass spectrometer (Waters). Metabolites were separated on a Waters BEH C18 column (100 × 2.1 mm, 1.7 μm) at 30 °C using a gradient of 0.1% formic acid in water (A) and 0.1% formic acid in acetonitrile (B): 0–1 min, 2–5% B; 1–4 min, 5–90% B; 4–8 min, 90% B; 8–10 min, 90–100% B; 10–12 min, 100–2% B. The flow rate was 0.3 mL min^−1^. The MS system was operated in the ESI^ + ^and ESI^−^ modes. The mass range was set at m/z 50–1000 Da in the full-scan mode. The optimized ESI parameters were: capillary voltage, 3.0 kV; cone voltage, 35 V; source temperature, 100 °C; desolvation gas temperature and flow, 350 °C and 800 L h^−1^, respectively. Leucine-enkephalin was used as the lock spray standard ([M + H]+ = 556.2771; [M − H]− = 554.2615) for accurate mass measurement at a concentration of 50 pg μL^−1^ and flow rate of 50 μL min^−1^. UHPLC-TOF/MS provided mass accuracy over 1 ppm (with lock mass calibration) and MS resolution over 40,000 FWHM in the V mode.

### Data processing

MS data were imported into Progenesis QI V2.0 (Nonlinear Dynamics, Newcastle, UK) within the MassLynx software (version 4.1) for peak detection and alignment. Peak detection was performed across the mass range of m/z 100–1000 with the retention time (RT) between 0.5 and 12 min, intensity threshold of 100, mass window of 0.02, RT window of 0.1, and noise elimination of 6.0; peak widths were detected automatically. The data were normalized to the total ion intensity per chromatogram, and loaded into the EZinfo 3.0 software for unsupervised principal component analysis (PCA) to obtain a general overview of metabolic phenotypes. Features showing an insignificant difference between TF-treated and control yeasts were filtered out using multivariate analyses, orthogonal partial-least-squares discrimination analysis (OPLS-DA) was performed for supervised analysis to identify features with discriminative power. A score plot was applied to reduce the dimensionality of the data for grouping of the samples, in which each point in the score plot represented the individual sample, and similar data sets exhibited clustering, while different sets separated farther apart. OPLS-DA models were validated based on accuracy, the multiple-correlation coefficient (*R*^2^), and cross-validated *R*^2^ (*Q*^2^) in cross-validation. Loading plots were generated from OPLS-DA and showed the impact of variables on vector formation. The combined S-plots and variable importance in the projection (VIP > 1) plots from the OPLS-DA were used to select distinct variables as potential biomarkers^49^.

### Metabolite identification

Features with significant differences were selected for metabolite identification. The MS/MS spectra of the potential biomarkers and commercially available reference standards were processed using the Waters MassLynx software version 4.1. Potential molecular formulas based on the accurate mass and isotopic pattern recognitions of parent and fragment ions were generated. All putative identities were confirmed by matching with entries in the ChemSpider (http://www.chemspider.com/), Human Metabolome Database (HMDB) (http://www.hmdb.ca/), METLIN (http://metlin.scripps.edu/), MassBank (http://www.massbank.jp/), LipidMaps (http://www.lipidmaps.org/), and KEGG (http://www.genome.jp/kegg) databases using exact molecular weights, nitrogen rule, or MS/MS fragmentation pattern data and a literature search. Efforts were made to distinguish metabolites from other isobaric compounds whenever possible based on elution order and degree of difference in fragmentation patterns corresponding to their structural characteristics. The putative identities of the biomarkers were confirmed by comparing their RTs and MS/MS spectra with those of obtained standards. Finally, identified differential metabolites were subjected to pathway topology analysis by MetaboAnalyst 3.0^46^.

### Statistical analysis

The data were expressed as the means ± standard errors of biological triplicates. REGTOX (http://eric.vindimian.9online.fr/en_index.html), which models the dose-response relationship with the non-linear Hill equation, was used to calculate inhibitory concentrations in the *S. cerevisiae* growth bioassays. Differences in amino acids, sugars, and higher alcohols and esters measured in beer were determined by one-way ANOVA. The raw data obtained by using the ET and EN were analyzed by unsupervised pattern recognition techniques with PCA. PCA can be done by eigenvalue decomposition of a data covariance matrix or singular value decomposition of a data matrix. The first PC has the largest possible variance, and each succeeding component has the highest variance possible under the constraint that it be orthogonal to the preceding components. The higher cumulative contribution rate is and the more original information will be reflected. Statistical analyses were carried out using the STATISTICA 7.0 software (StatSoft Inc., Tulsa, OK, USA), statistical significance was set at *P* ≤ 0.05.

## Additional Information

**How to cite this article**: Kong, Z. *et al*. The fungicide triadimefon affects beer flavor and composition by influencing *Saccharomyces cerevisiae* metabolism. *Sci. Rep.* **6**, 33552; doi: 10.1038/srep33552 (2016).

## Supplementary Material

Supplementary Information

## Figures and Tables

**Table 1:** Fermentable sugar content (g L^−1^) in beer samples with and without TF residues.

Compound	CK	Treatment
Fructose	ND	ND
Glucose	0.2 ± 2.2	0.2 ± 3.5
Sucrose	ND	ND
Maltose	0.1 ± 2.8	0.2 ± 3.1
Maltotriose	0.6 ± 3.6	1.8 ± 4.4*

*P < 0.05.

**Table 2:** Concentration (mg L^−1^) of higher alcohols and esters in beer samples with and without TF residues.

Compound		CK	Treatment
Higher alcohols	*n*-propanol	13.2 ± 2.6	12.9 ± 3.3
	*n*-Butanol	8.7 ± 4.8	8.6 ± 2.7
	Isobutyl alcohol	12.5 ± 1.8	9.8 ± 3.5*
	Isoamyl alcohols	52.6 ± 6.4	46.3 ± 5.9*
	β-Phenylethyl alcohol	32.3 ± 2.9	31.5 ± 4.6
Higher esters	Ethyl acetate	22.3 ± 3.6	34.5 ± 3.8*
	Ethyl butyrate	0.2 ± 1.5	0.2 ± 2.4
	Isoamyl acetate	2.8 ± 1.9	2.6 ± 1.6
	Ethyl caprylate	0.4 ± 2.2	0.3 ± 2.9
	Phenylethyl acetate	1.8 ± 4.5	1.6 ± 3.5
	Ethyl lactate	0.6 ± 1.7	0.6 ± 2.6
	Ethyl caproate	0.3 ± 2.3	0.2 ± 3.1

^*^P < 0.05.
