# Supplementary material for: The fungicide triadimefon affects beer flavor and composition by influencing Saccharomyces cerevisiae metabolism
Source: Sci Rep. 2016 Sep 15;6:33552. doi: 10.1038/srep33552 (PMC5024320; doi:10.1038/srep33552)
Supplement: Supplementary Information [file srep33552-s1.pdf]

[Supplementary material] including 5 figures and 4 tables

The fungicide triadimefon affects beer flavor and composition by influencing *Saccharomyces cerevisiae* metabolism

Zhiqiang Kong [a, b, 1], Minmin Li [a, 1], Jingjing An [c], Jieying Chen [a], Yuming Bao [a], Frédéric Francis [b], Xiaofeng Dai [a,*]

[a] Institute of Food Science and Technology, Chinese Academy of Agricultural Sciences/Key Laboratory of Agro-Products Processing/Laboratory of Agro-products Quality Safety Risk Assessment, Ministry of Agriculture, Beijing 100193, P. R. China

[b] Functional and Evolutionary Entomology, Gembloux Agro-Bio-Tech, University of Liège, Passage des Déportés 2, 5030 Gembloux, Belgium

[c] College of Food Science, Northeast Agricultural University, Key Laboratory of Dairy Science, Ministry of Education, Harbin 150030, China

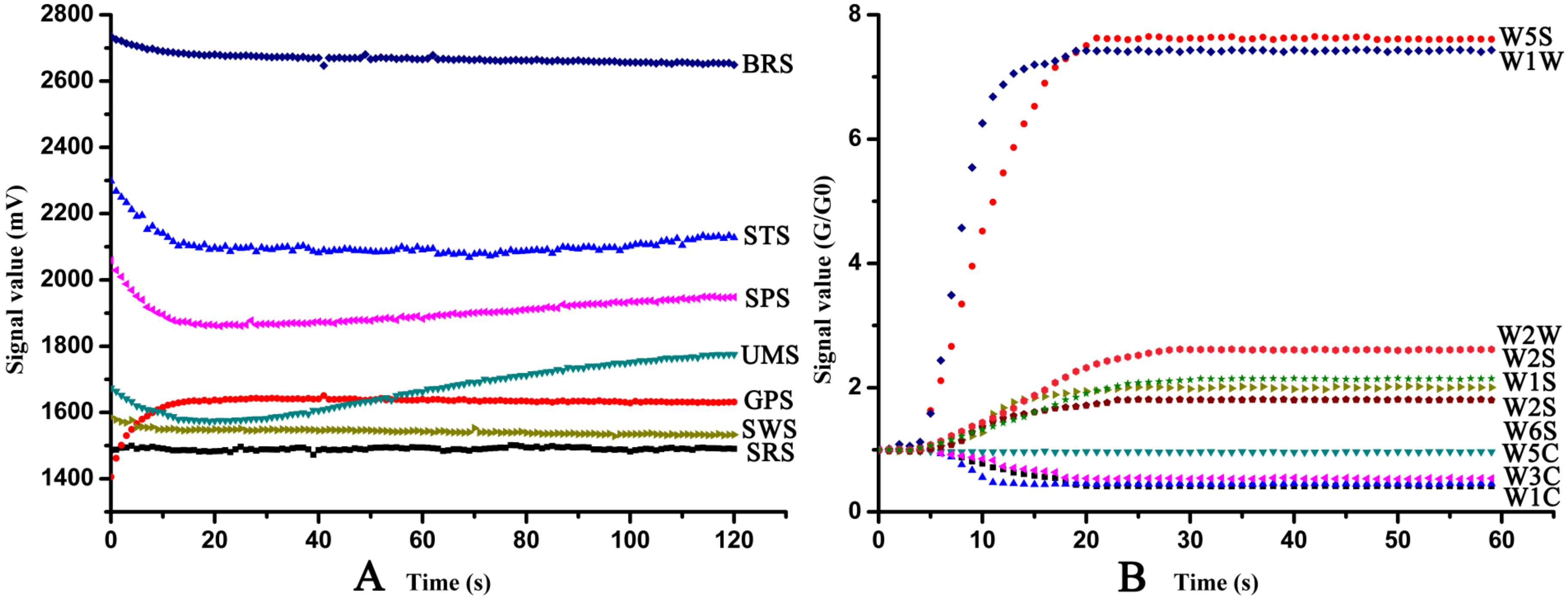

Fig. S1. Typical responses of the ET (A) and EN (B) to beer samples brewed in the presence or absence of TF.

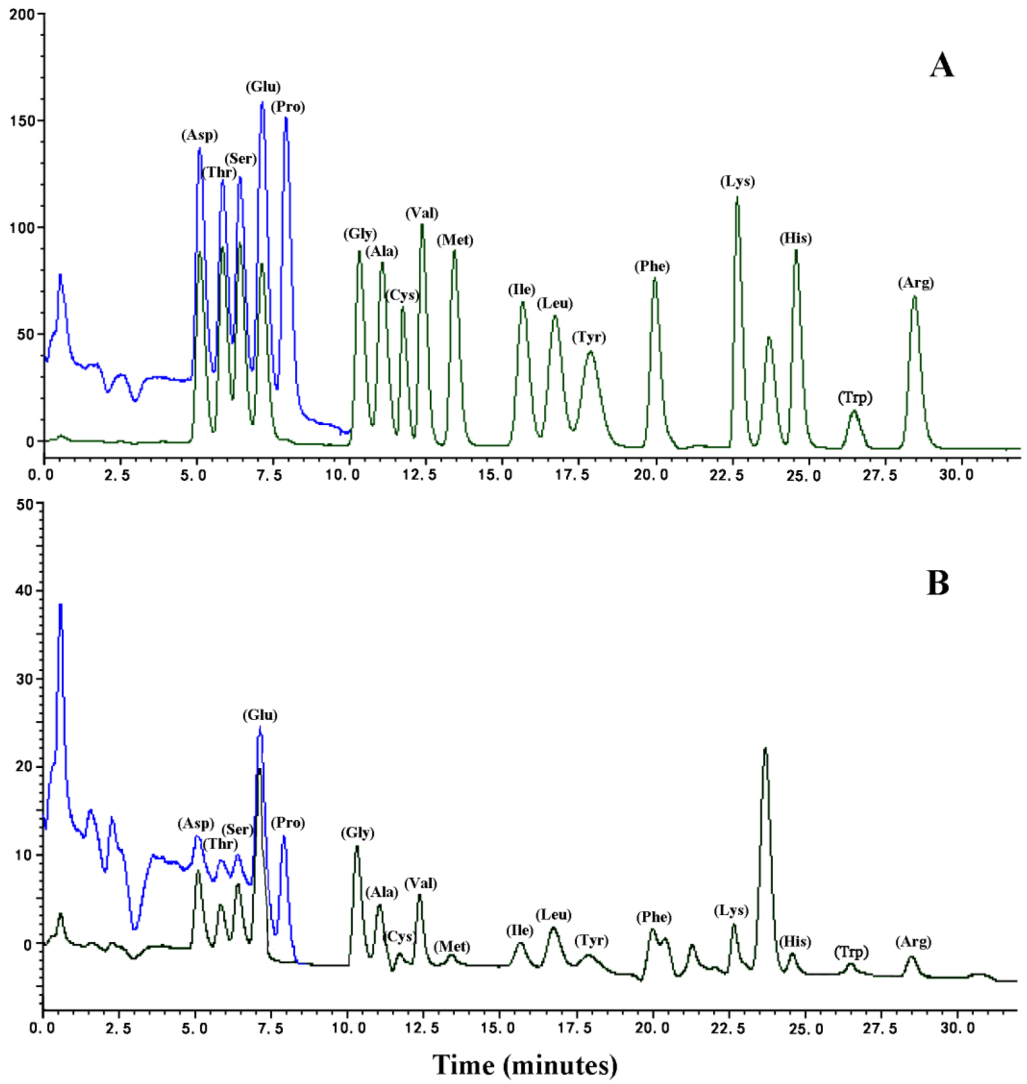

Fig. S2. Chromatograms of amino acid standards (A) and beer samples (B).

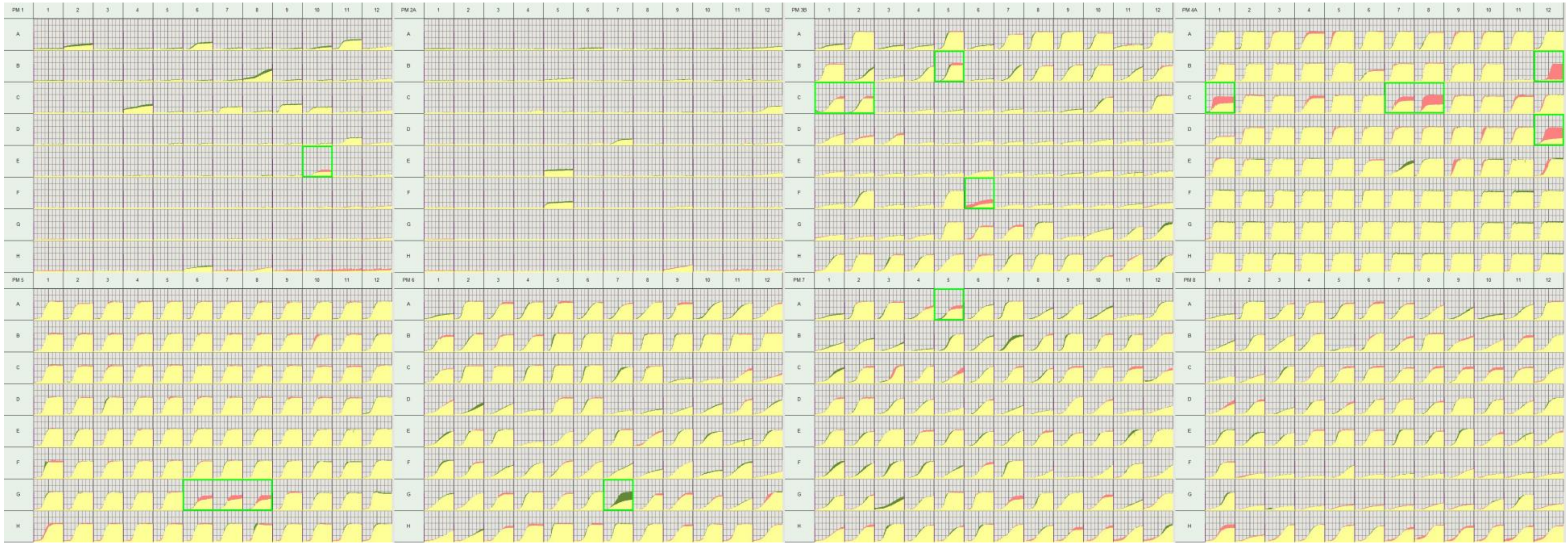

Fig. S3. Phenotype microarray analysis of *S. cerevisiae* incubated with TF. Phenotype microarray assay based on redox signal intensities showing untreated *S. cerevisiae* in pink and TF-treated *S. cerevisiae* in green; yellow indicates similar metabolic output.

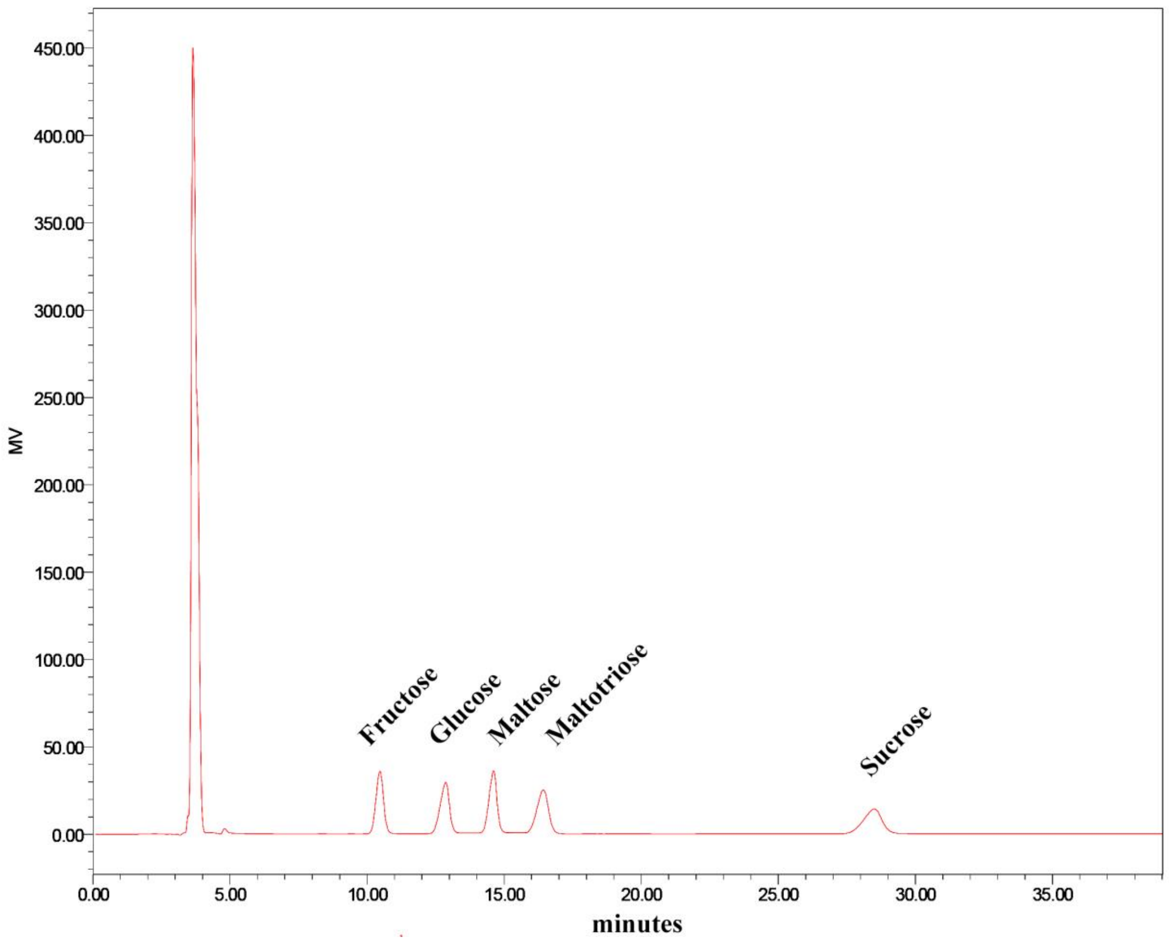

Fig. S4. HPLC chromatogram of standards: sucrose, glucose, fructose, maltose, and maltotriose.

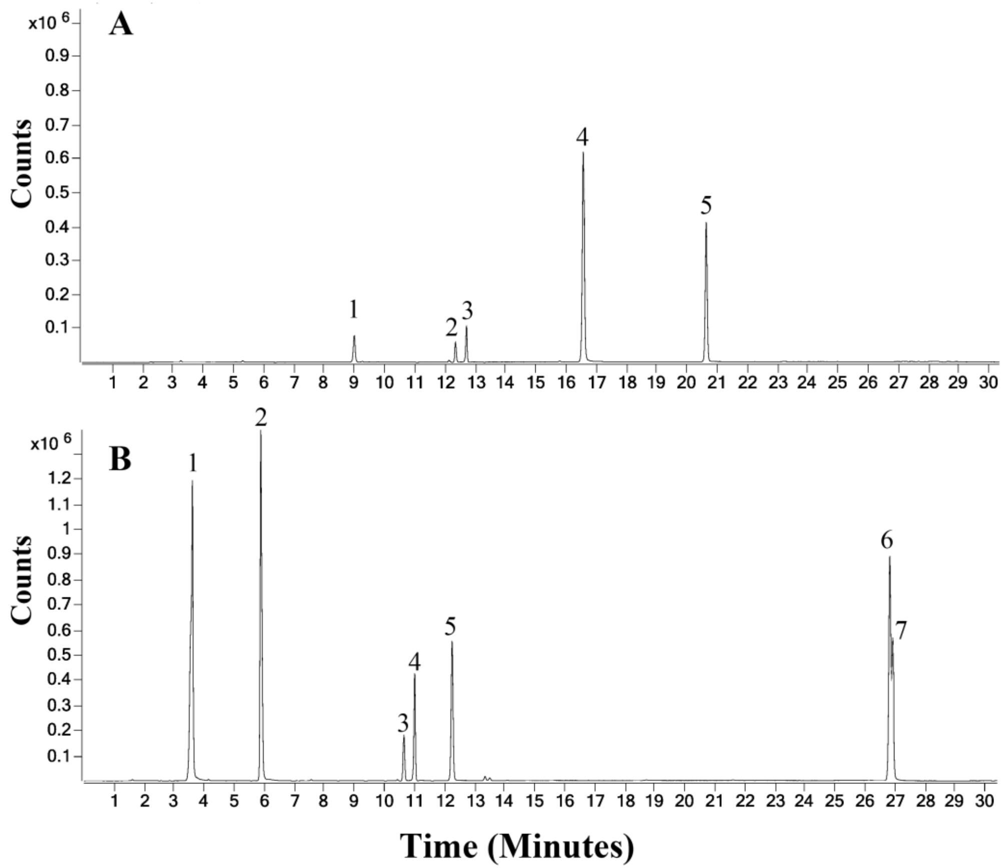

Fig. S5. GC-MS chromatogram of higher alcohol and ester standards. (A) Higher alcohols, 1, *n*-propanol; 2, *n*-Butanol; 3, Isobutyl alcohol; 4, Isoamyl alcohols; 5, β-Phenylethyl alcohol. (B) Higher esters, 1, Ethyl acetate; 2, Ethyl butyrate; 3, Isoamyl acetate; 4, Ethyl caprylate; 5, Phenylethyl acetate; 6, Ethyl lactate; 7, Ethyl caproate.

Table S1. Relative standard deviations and one-way ANOVA of sensory evaluation of beer samples.

Sensors	Control (%)	Treatment (%)	*P* (one-way ANOVA)
SRS	8.24	2.42	<0.05
GPS	6.93	1.68	<0.05
STS	1.81	13.24	<0.05
UMS	0.58	6.79	<0.05
SPS	6.39	4.45	<0.05
SWS	0.54	11.22	<0.05
BRS	4.95	9.25	<0.05
W1C	14.21	5.67	<0.05
W5S	6.36	3.98	<0.05
W3C	1.54	12.62	<0.05
W6S	10.81	8.74	<0.05
W5C	3.08	10.51	<0.05
W1S	13.94	0.68	<0.05
W1W	7.02	4.42	<0.05
W2S	12.59	3.89	<0.05
W2W	10.35	5.67	<0.05
W3S	5.45	11.36	<0.05

Table S2. Metabolic profiles of *S. cerevisiae* treated or not with TF.

Mode	ID	Formula	Description	m/z	Retention time (min)	Max fold change *
	1	$C_{25}H_{47}NO_5$	3-Hydroxy-9Z-octadecenoylcarnitine	480.3084	4.64	7.12
	2	$C_{28}H_{42}O_5$	Pubesenolide	458.3032	6.27	4.27
	3	$C_{26}H_{52}NO_7P$	LysoPC (18:1(9Z))	522.3567	5.08	3.26
ES^+ upregulated	4	$C_{35}H_{38}C_{l2}N_8O_5$	Hydroxyitraconazole	759.1948	11.34	1.98
	5	$C_{21}H_{40}O_4$	Heneicosanedioic acid	339.2896	5.07	2.58
	6	$C_{19}H_{36}O_4$	Avocadene 1-acetate	311.2583	4.53	3.17
	7	$C_8H_{20}NO_6P$	Glycerophosphocholine	296.0662	1.76	2.27
	8	$C_8H1_4N_2O_5S$	γ-Glutamylcysteine	268.1041	0.98	3.75
	9	$C_{29}H_{46}O$	Delta 8,14-Sterol	411.3595	8.17	25.81
	10	$C_{26}H_{52}NO_7P$	LysoPC (18:1(9Z))	522.3567	5.08	3.27
	11	$C_{29}H_{48}O$	4a-Methylfecosterol	395.3666	9.46	12.25
	12	$C_{28}H_{44}O$	Ergosterol	379.3355	7.89	1.23
	13	C_8H_8O	Phenylacetaldehyde	184.0738	5.08	3.69
ES^+	14	$C_6H_9N_2O_5P$	Imidazole acetol-phosphate	111.0202	11.39	1.45
downregulated	15	$C_{10}H_{12}N_2O_3$	L-Kynurenine	250.1189	4.48	1.42
	16	$C_6H_{11}NO_4$	Aminoadipic acid	162.0758	0.96	3.39
	17	$C_{18}H_{39}NO_3$	Phytosphingosine	318.3006	4.24	1.52
	18	$C_{16}H_{32}O_2$	Palmitic acid	274.2745	3.90	1.65
	19	$C_{11}H_{15}N_5O_3S$	5'-Methylthioadenosine	298.0972	2.52	2.76
	20	$C_{24}H_{50}NO_7P$	LysoPC (16:0)	496.3406	5.08	2.59
	21	$C_{35}H_{67}O_{13}P$	PI (12:0/14:0)	725.4251	8.69	8.30
	22	$C_{20}H_{38}O_7S$	2-Methylacetophenone	421.227	10.18	5.96
ES^- upregulated	23	$C_{13}H_{14}O$	(*E*)-2-Tridecene-4,6,8-triyn-1-ol	371.2017	6.31	4.13
	24	$C_{19}H_{19}N_3O_5S$	Oxacillin	446.1023	9.20	3.46
	25	$C_{51}H_{99}O_{13}P$	PI (21:0/21:0)	949.6751	3.01	1.92
	26	$C_{16}H_{28}N_2O_2$	Phygrine	325.2139	5.66	2.03
ES^-	27	$C_{10}H_{17}N_3O_6S$	Glutathione	306.0759	0.97	1.64
downregulated	28	$C_{12}H_{22}O_{11}$	Sucrose	341.1085	0.81	2.45

29	$C_{10}H_{14}N_5O_7P$	2'-Deoxyguanosine 5'-monophosphate	346.0554	0.93	1.95
30	$C_{29}H_{46}O_3$	4a-Carboxy-4b-methyl-5a-cholesta-8,24-dien-3b-ol	423.3258	7.67	1.78
31	$C_9H_{13}N_3O_5$	Cytidine	242.079	0.77	3.54

* The fold change refers to the treated vs. the untreated group

Table S3: Linearity, quantification limits, recovery, repeatability, and reproducibility of the sugar determination method.

Compound	Concentration range (g/L)	R^2	LOQ (mg/L)	Recovery [a] (%)	RSD [b] (Repeatability, %)	RSD [c] (Reproducibility %)
Fructose	0.1-10	0.9999	2.0	95	2.1	6.6
Glucose	0.1-10	0.9962	3.0	98	1.2	1.9
Sucrose	0.1-10	0.9979	2.0	101	4.2	2.8
Maltose	0.1-10	0.9985	3.0	92	3.5	4.9
Maltotriose	0.1-10	0.9991	5.0	94	8.6	5.4

a: For recovery calculations, beer samples were spiked at 100 mg L^{-1} for all sugars
b: n = 10
c: n = 5

Table S4: Linearity, quantification limits, recovery, repeatability, and reproducibility of the higher alcohol and ester determination method.

Compound		Concentration range (μg/L)	R^2	LOQ (μg/L)	Recovery [a] (%)	RSD [b] (Repeatability, %)	RSD [c] (Reproducibility %)
Higher alcohols	*n*-propanol	500-10000	0.9986	5.0	85	4.4	5.4
	n-Butanol	500-10000	0.9973	5.0	90	3.9	12.3
	Isobutyl alcohol	500-10000	0.9980	10.0	88	5.7	1.8
	Isoamyl alcohols	500-10000	0.9981	5.0	95	5.8	9.6
	β-Phenylethyl alcohol	500-10000	0.9951	10.0	106	2.5	5.5
Higher esters	Ethyl acetate	100-10000	0.9924	1.0	92	5.0	7.2
	Ethyl butyrate	100-10000	0.9990	1.0	95	11.8	9.0
	Isoamyl acetate	100-10000	0.9966	2.0	83	2.1	6.2
	Ethyl caprylate	100-10000	0.9975	5.0	101	7.4	6.8
	Phenylethyl acetate	100-10000	0.9989	2.0	91	4.9	13.6
	Ethyl lactate	100-10000	0.9949	10.0	109	8.6	6.1
	Ethyl caproate	100-10000	0.9992	5.0	93	3.3	4.8

a: For recovery calculations, beer samples were spiked at 1000 μg L^{-1} for all higher alcohols and higher esters

b: n = 10

c: n = 5

Method validation of beer organic contents determination

Identification and quantification of sugars, higher alcohols and esters was based on their retention times, and the peak areas were compared against the standard calibration curves. Typical chromatograms are shown in Figs. S4 and S5. As shown in the Tables S3 and S4, linearity was evaluated using standard solutions, in the range of 0.1–10 g L^{-1} for sucrose, glucose, fructose, maltose, and maltotriose, 500–10,000 μg/L for higher alcohols and 100–10000 μg L^{-1} for higher esters. Linearity was observed for all the target compounds, with R^2 values greater than 0.9924. The limits of quantitation (LOQs) for five sugars ranged from 2.0–5.0 mg L^{-1}, and the LOQs of higher alcohols and esters were in the range of 1.0 to 10.0 μg L^{-1}. The recoveries of five sugars in the beer matrix were within 92–101% at the tested concentrations, and those of higher alcohols and esters were within 83–109%. Moreover, the proposed method showed good repeatability (n = 5) and reproducibility (n = 10), with relative standard deviations ranging from 1.2 to 13.6%. The recovery assay results illustrate that the method used to determine sugars and higher alcohols and esters in beer has good precision and accuracy.
